# Supplementary material for: Subslab ultra low velocity anomaly uncovered by and facilitating the largest deep earthquake
Source: Nat Commun. 2024 Mar 29;15:2754. doi: 10.1038/s41467-024-47129-3 (PMC10980710; doi:10.1038/s41467-024-47129-3)
Supplement: Supplementary file 1 — Supplementary Information [file 41467_2024_47129_MOESM1_ESM.pdf]

Supplementary Information for

Subslab ultra low velocity anomaly uncovered by and facilitating the largest deep earthquake

Weiwen Chen[1,2], Shengji Wei[*1,3,4] & Weitao Wang[*2]

[1]Earth Observatory of Singapore, Nanyang Technological University, Singapore.

[2]Institute of Geophysics, China Earthquake Administration, China.

[3]Asian School of the Environment, Nanyang Technological University, Singapore.

[4]Institute of Geology and Geophysics, Chinese Academy of Sciences, China.

*Corresponding author: shjwei@ntu.edu.sg; wangwt@cea-igp.ac.cn

This file includes:

Supplementary Notes 1 to 2

Supplementary Figures 1 to 16

Supplementary Tables 1 to 2

Supplementary References

Supplementary Note 1. Station calibration

To identify clean paths at the receiver-side for RSFT modeling, we employ a path calibration technique that involves modeling a smaller nearby earthquake with a magnitude of Mw6.7 using the Cut-And-Paste (CAP) method[1]. This calibration event, with a relatively short duration of approximately 1.5 seconds[2], is considered as a point source at 2 seconds and longer periods.

To simulate the observed P-waves, we utilize teleseismic stations and generate 1-D synthetic waveforms based on the PREM (Preliminary Reference Earth Model) [3], specifically targeting the frequency range of 0.1 to 0.5 Hz. The modeling results, presented in Supplementary Figure 2, demonstrate good agreement between the 1-D synthetic waveforms and the data. However, certain stations within the EU array exhibit deviations, which may be attributed to the presence of the slab structure near the earthquake source and the complex upper mantle structure beneath the EU array. Conversely, the fits for other arrays such as WA and CN indicate that the 1-D synthetic waveforms can serve as reliable Empirical Green's Functions (EGF) for modeling the subevents of the mainshock in these arrays. Consequently, we select the stations with good waveform fits for further investigation and analysis in the mainshock study.

Supplementary Note 2. Discussion on potential factors may influence the deconvolution results

a. 3-D out-of-plane multipathing

Multipathing can arise from both in-plane 2-D heterogeneity and out-of-plane 3-D effects[4,5]. By examining distance and azimuthal record sections (Figure 3), we observe that the multipathing contrast between S2 and S1 is more pronounced in the distance profiles rather than the azimuthal profiles, indicating that the dominant multipathing is in-plane. However, MPD analysis reveals strong out-of-plane multipathing around the 100°W longitude in the USArray (Figure 6c), which is partly captured by the reduction in SULVA thickness from the WA to EA array (Figure 9e).

Investigating the detailed 3-D structure of SULVA and assessing its effects on multipathing is beyond the scope of this paper as it is limited by the aperture of the seismic arrays. However, we can still verify whether a 3-D structure with smaller velocity perturbations but larger dimensions can produce the observed multipathing through 3-D synthetic simulations. To generate high-frequency synthetic waveforms, we employed a hybrid numerical method[6] and computed waveforms at 0.8 Hz to generate 3-D synthetic waveforms.

To achieve this objective, two 3-D models were developed: (a) An 80-km thick slab model with a symmetric positive velocity perturbation centered at the core of the subduction, having a dipping angle of 45° and penetrating through the 660-D discontinuity.(b) A parallelepiped-shaped 3-D low velocity zone (LVZ) was positioned along the teleseismic ray path beneath the seismic source. The LVZ had sharp boundaries designed to generate multipathing effects within a specific distance range. The cross-section of the LVZ exhibits a

parallelogram shape, with a boundary length of approximately 70 km along the ray path and about 30 km between the upper and lower boundaries (Supplementary Figure 9b). Additionally, there is a 50 km extension perpendicular to the cross-section.

By calculating the wavefields at the boundaries of the box containing the 3-D LVZ structure, the Direct Solution Method (DSM) was employed to generate synthetic displacements at teleseismic distances[7,8]. The same deconvolution approach used for the 2-D synthetics was applied to derive the RSTF from the 3-D synthetics. The results indicate that the slab model, featuring a maximum velocity perturbation of 5% and penetrating through the 660-D discontinuity, only slightly broadens the RSTF and does not produce a clear multipathing "double-peak" associated with multipathing. In contrast, the 3-D SULVA model incorporating an 18% reduction in P-wave velocity successfully generates the expected multipathing waveforms within the anticipated distance range (Supplementary Figure 9).

b. Attenuation

To generate the Green's functions, we employed a 1-D velocity model with moderate mantle attenuation. However, it is crucial to consider the impact of upper mantle attenuation, as it can significantly alter seismic waveforms, particularly at high frequencies[9]. To investigate this effect, we tested three models with different attenuation levels: minimal attenuation (Qp(min) = 20000, Qs(min) = 10000), moderate attenuation (Qp(min) = 320, Qs(min) = 160), and strong attenuation (Qp(min) = 160, Qs(min) = 80). To assess the impact of attenuation, we placed a source with a duration of 0.5 s at a depth of 600 km and generated synthetic waveforms at teleseismic distances using the different attenuation models (green waveform in Supplementary Figure 10a). These synthetics were treated as Empirical Green's Functions (EGF). Additionally, we generated three sets of synthetics using the same 1-D models but with a longer source duration of 2 seconds, which served as the "data" waveforms. We then applied the PLD method between the three data sets and the three EGF sets to obtain nine RSTFs at frequencies ranging from 0.2 to 0.8 Hz. Supplementary Figure 10 demonstrates that when the synthetic data with no attenuation was deconvolved by the high-attenuation EGF, strong artifacts were observed in the RSTF, which could lead to issues such as phase misidentification. However, in all other scenarios, the RSTFs exhibited consistent durations compared to the input waveforms (Supplementary Figure 10). It is important to note that none of these RSTFs exhibited the multipathing feature observed by the North American stations. This analysis underscores the importance of accounting for upper mantle attenuation when analyzing seismic waveforms, especially at higher frequencies. While the RSTF analysis demonstrates the impact of attenuation on waveform characteristics, the absence of multipathing features in the RSTFs suggests that factors other than attenuation might be responsible for the observed

multipathing phenomenon observed by the North American stations. Further investigation is needed to fully understand and explain the mechanisms behind these observations.

c. Anisotropy

In alignment with a recent study by Li et al.[10], which highlights the significant impact of strong anisotropy on deep intra-slab earthquakes, we conducted tests to assess the influence of anisotropy on RSTF waveforms using 3-D synthetics[11]. To simulate the anisotropic structure observed by Li et al., we incorporated a 10 km-thick anisotropy layer on top of a 90 km-thick slab (Supplementary Figure 11). By adjusting the amplitude of anisotropy, as represented by the ratio of anisotropy parameters pairs [12], we evaluated the effects on the waveforms. The results demonstrate that at frequencies of 0.01-0.1Hz and 0.1-0.5 Hz, teleseismic waveforms are not significantly impacted when the anisotropy is weak. Although some polarity flipping is observed at stations aligned with the nodal direction as the anisotropy strength increases (Supplementary Figure 11), these minor waveform distortions are inadequate to explain the observed strong multipathing phenomenon.

d. Variation on dlnVs/dlnVp

Deep earth interior often exhibits extreme velocity reductions, which are indicative of partial melting [13,14]. In partially melted rocks, the ratio of fractional changes between Vs and Vp (dlnVs/dlnVp) is typically higher compared to non-melted rocks[15]. The pore geometry of the partially melted material plays a crucial role in determining the Vp/Vs ratio, and dlnVs/dlnVp can vary from 0.6 to 2.3 depending on the aspect ratio of the pore geometry [15–17]. However, in this study, we are unable to estimate the pore geometry at the bottom of the upper mantle since its relationship with shear velocity reduction is not well understood. To explore the impact of different velocity fractional change ratios, we generated 2-D synthetic P waveforms under varying values of dlnVs/dlnVp ranging from 1.6 to 2.2 (Supplementary Figure 12). We observed that the P waveform in the initial 10 seconds remained nearly identical across all teleseismic distances. Consequently, in this study, we focused on using the initial portion of the first P-arrival, which is expected to be less influenced by variations in the Vp/Vs ratio.

e. Variation of focal mechanism

Radiation pattern analysis indicates a general consistency in the focal mechanisms (FM) of the subevents throughout the entire rupture process of the 2013 Sea of Okhotsk earthquake[18]. However, the results of multiple point source inversion reveal considerable variations, particularly during the initial stage of the rupture[19]. Although the dominant features of the FM align with normal faulting, there are noteworthy discrepancies. To assess the influence of FM variation on the deconvolution results, we conducted a study using synthetics generated from the globalCMT solution as the EGF. Pseudo-data comprising two subevents were

created: the first subevent involved a 10° steeper dip rotation (at 0 seconds), while the second subevent maintained the original globalCMT FM (at 3 seconds) as a reference. RSTFs derived after deconvolution indicated that the first subevent was minimally affected across most of the focal sphere. Notably, variations were observed near the nodal plane, primarily influencing amplitude changes. However, no significant multipathing effects were observed in the distance profile (Supplementary Figure 13).

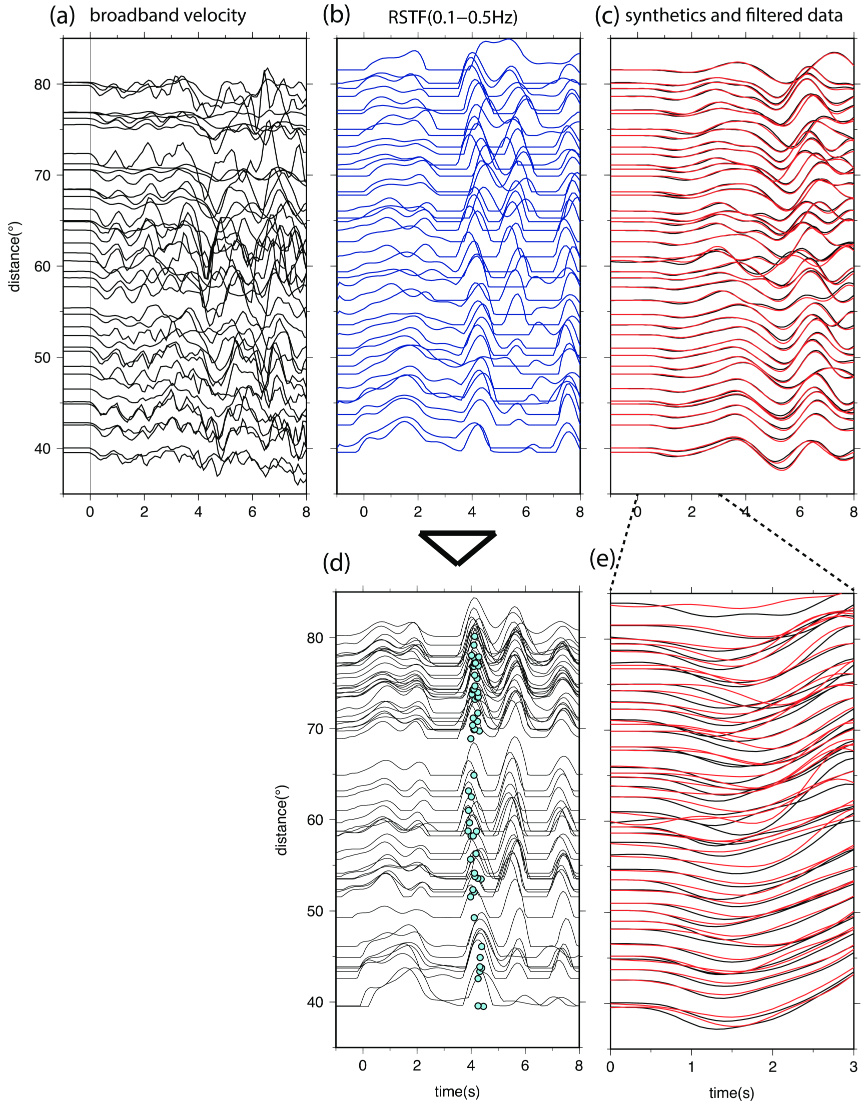

Supplementary Figure 1 | The application of Projected Landweber deconvolution (PLD) to the initial 10 seconds of velocity records obtained from the stations of the EU Array, using a bandpass filter of 0.1-0.5 Hz. (a) Original velocity records, with teleseismic P onset manually picked at 0 seconds. For clarity, only one

station within a 1-degree distance is plotted. (b) Relative source time functions (RSTFs) retrieved after deconvolution using corresponding 1-D synthetics. (c) Waveform fitting between the filtered original data (black) and synthetic waveform derived by convolving RSTFs and 1-D synthetics. (d) Stacked RSTFs waveform (referred to in section 2.1), with subevent s2 peaks marked with cyan dots. (e) Zoom-in features of subplot (c).

During the PLD process, constraints (positive, causal, and bounded) are imposed on the deconvolution iterations, guided by the physical characteristics of the RSTFs[20]. The advantage of the PLD method over the traditional "water-level deconvolution" method is the clear distinction of the source energy signal without introducing negative artifacts from over-fitting. The synthetics derived from convolving the RSTF with EGF show good fitting to the data, except for the beginning part, which leads to a positive artifact before the 0 mark on the time axis. However, this artifact does not affect the measurement of the time delay between the onset and S2, which serves as an indicator of slab effect in subsequent analysis. The deconvolution is performed with a bandpass filter between 0.1 and 0.5 Hz. This filter range is chosen because the source time functions derived under this bandpass exhibit a series of coherent and distinct subevents, as clearly identified in Figure 2 and S3.

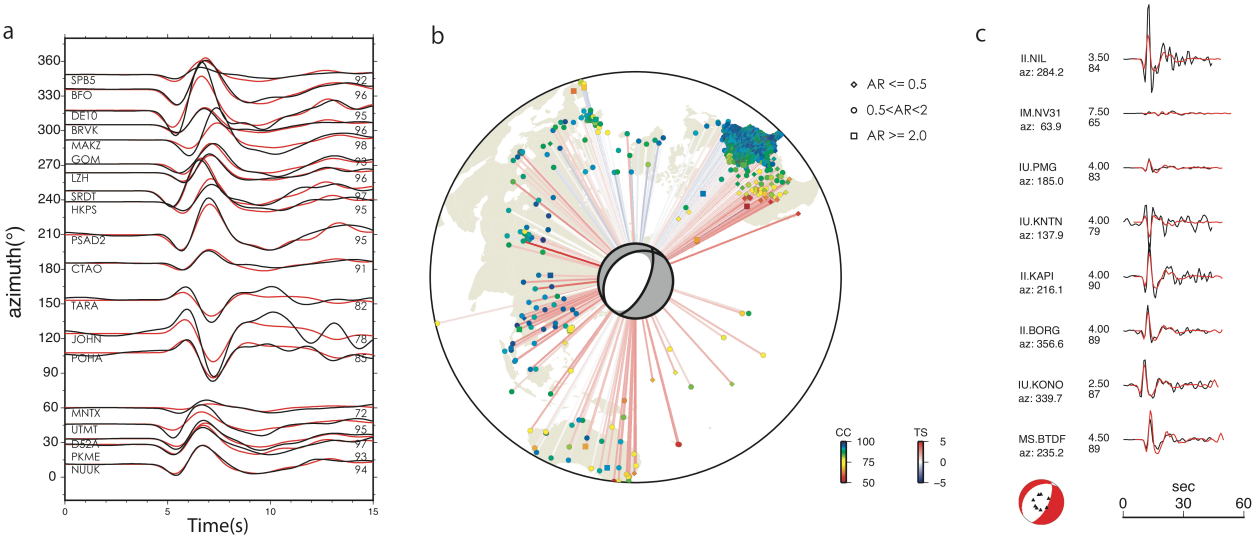

Supplementary Figure 2 | Using Mw 6.7 earthquake as a calibration event to choose the stations applied in the deconvolution for the Mw 8.3 main shock. (a) Representative 1-D waveform modeling for the teleseismic P wave of the Mw 6.7 aftershock, indicated by the beachball symbol in (b). Station names are listed on the left, and the cross-correlation coefficient (CC) is indicated on the right. (b) 1-D waveform modeling spidergram with the focal mechanism from globalCMT at the center. The quality of P wave modeling is evaluated based on the amplitude ratio (AR) represented by symbol shape, CC indicated by symbol color, and time shift (TS) between the data and synthetics depicted by line color. The modeling is conducted using the Cut-And-Paste method[1] within the frequency range of 0.1-0.5 Hz, which aligns with the frequency range employed in PLD in this study. (c) An example of the Cut-And-Paste method applied to the Mw 6.7 aftershock. The black and red waveform traces represent the data and synthetics, respectively. TS values are shown above the seismograms, and the CC are shown below. The projection of stations for the focal mechanism is displayed on the beach ball diagram below the seismograms.This analysis aids in determining the stations to be utilized in the deconvolution process, ensuring the inclusion of appropriate stations based on waveform modeling and quality evaluation.

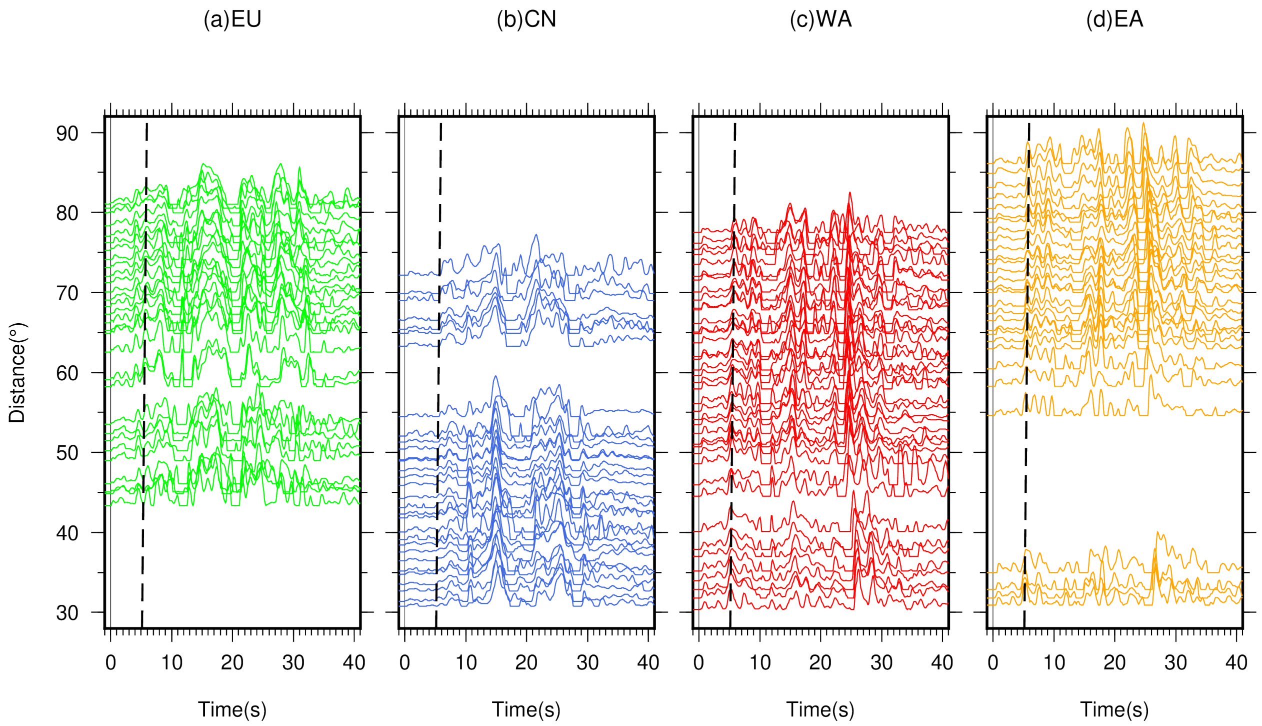

Supplementary Figure 3 | Distance profile of Relative Source Time Functions (RSTFs) for four arrays showing the complete wave train. The RSTFs are plotted as profiles of distance for each array. Dashed lines are plotted to indicate the arrivals of the reference S2 phase

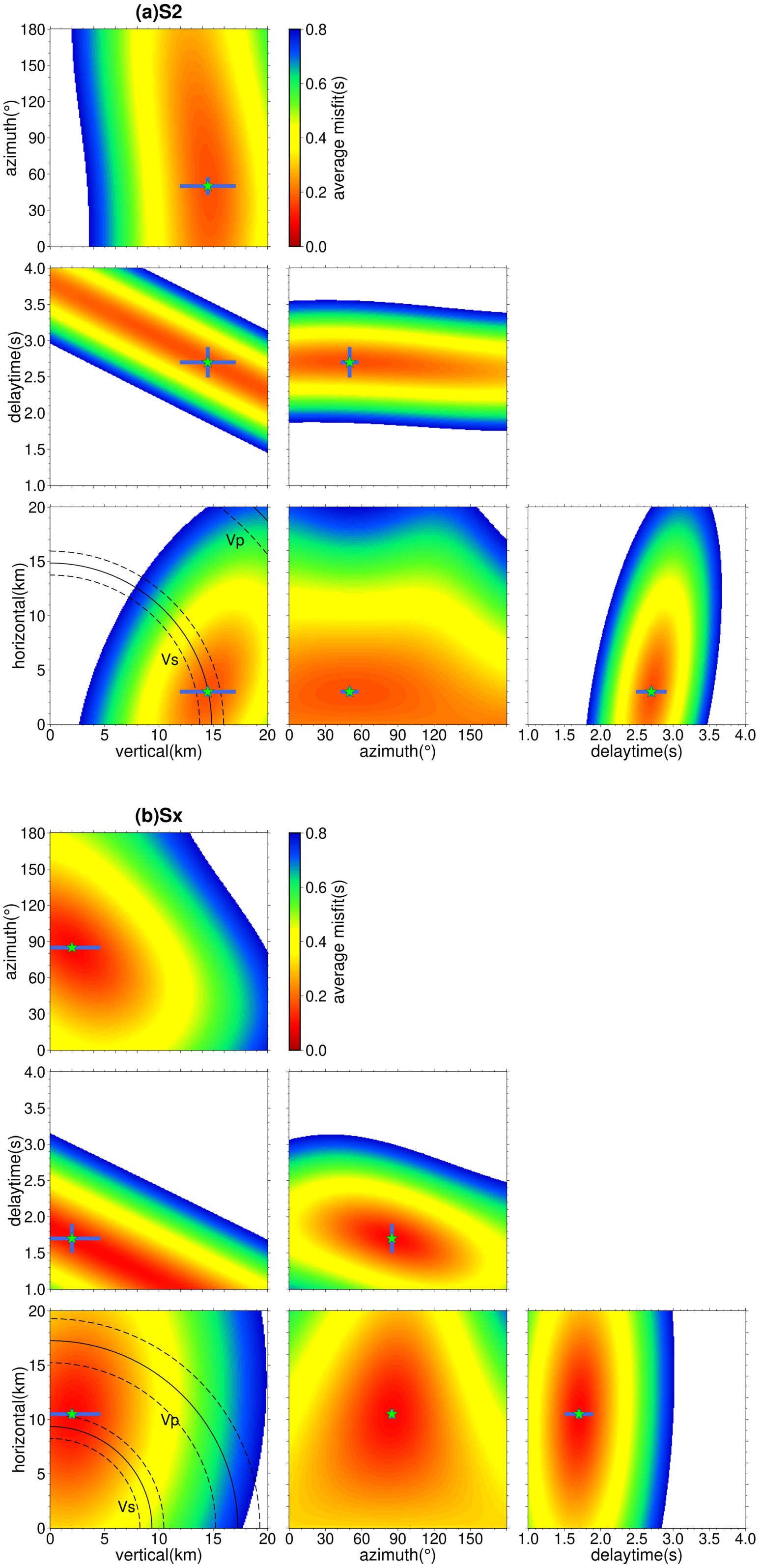

Supplementary Figure 4 | Trade-off sensitivity tests for 6 pairs of inverted parameters. (a) Subevent S2. (b) Subevent Sx. Solid contours illustrate the resulting rupture speed corresponding to the parameter

pair, while dashed contours represent the range of uncertainties associated with the inversion. The uncertainties were estimated through 1000 bootstrapping iterations, randomly selecting 90% of the stations.

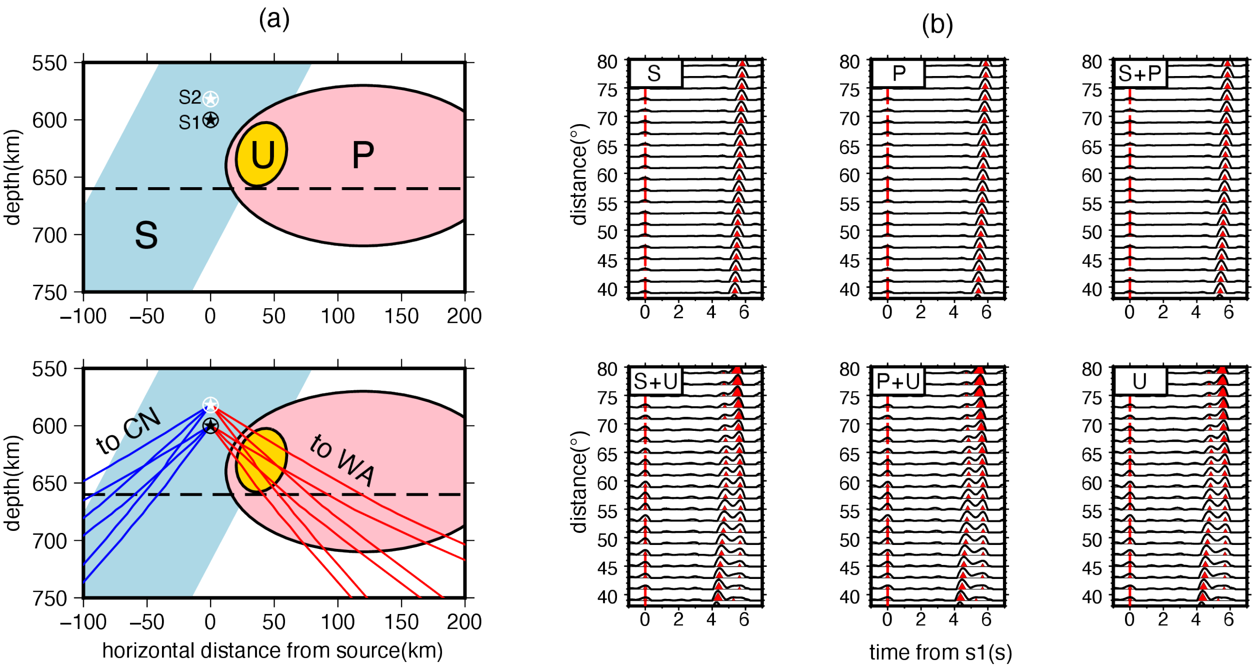

Supplementary Figure 5 | (a) Configuration of the "SUP" 2-D model and 1-D ray path depicting subevents S1 and S2 at the source region, with the 660 km discontinuity highlighted by dashed lines. The red and blue solid-dashed-solid curves represent the teleseismic P-wave ray paths, originating from the source and extending towards the WA3 and CN arrays, respectively, for distances of 40°, 60°, and 80° as predicted by the PREM earth model. A black star enclosed within a circle denotes subevent S1, whereas a white star represents S2. (b) RSTFs obtained from synthetic waveforms in the direction of the WA3 array using various combinations of "SUP" structures. The labels "S," "U," and "P" correspond to the slab structure, SULVA, and elliptical plume head structure, respectively. The slab structure exhibits a positive P-wave velocity anomaly with a maximum perturbation of 5% at its center, decreasing linearly towards its edges. Subevents S1 and S2 are located within the slab structure. The SULVA represents a uniform velocity perturbation, while the elliptical plume head structure (labeled as "P") is positioned across the 660 km discontinuity and demonstrates a peak velocity reduction of -3% at the center of the ellipse.

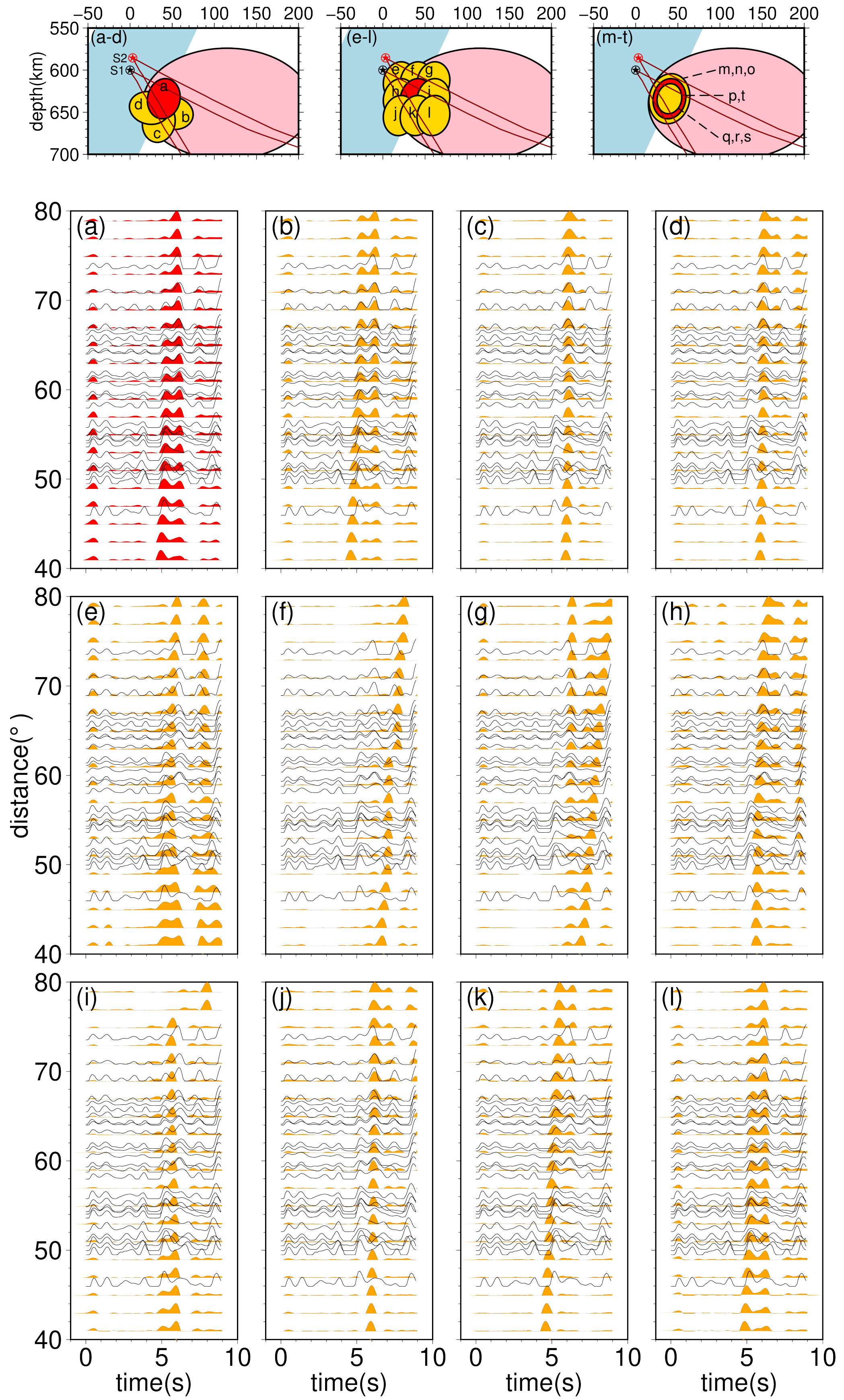

(a-d)
(e-l)
(m-t)
S2
S1
a
b
c
d
e
f
g
h
i
j
k
l
m,n,o
p,t
q,r,s
depth(km)
(a)
(b)
(c)
(d)
(e)
(f)
(g)
(h)
(i)
(j)
(k)
(l)
distance(°)
time(s)

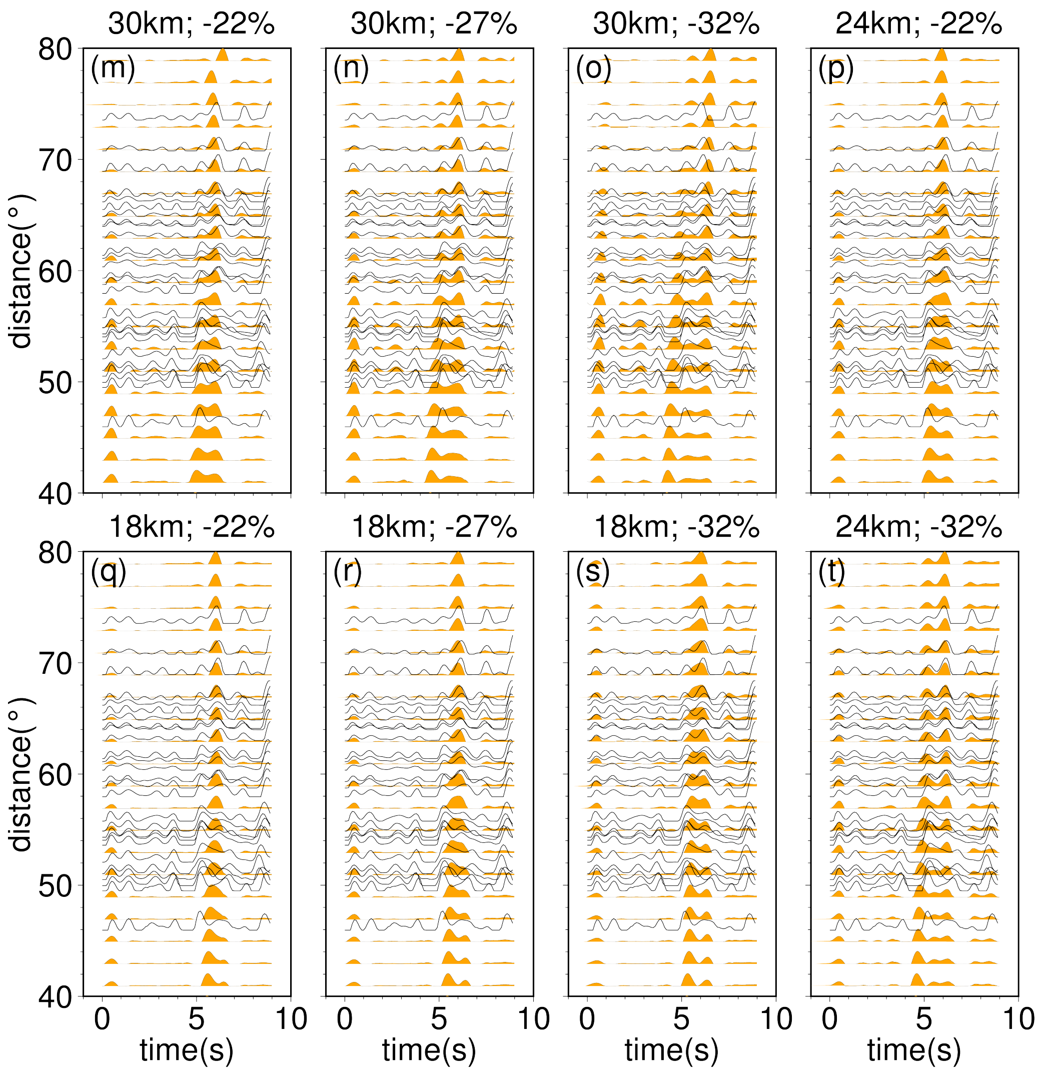

Supplementary Figure 6 | Top panels show the configuration of SUP models, featuring the relative positions of the SUP structures in relation to the subevent S1 and S2. The cyan and pink shaded regions represent the slab and plume, respectively. The dark red curves represent the range of teleseismic ray paths predicted by the PREM model. The red egg area denotes the spatial position of the optimal SULVA structure. The alternative positions and structures of SULVA are presented by golden eggs: (a-d) rotated by 90°, 180°, and 270° while maintaining fixed lower foci; (e-l) grid offset of 20 km in both horizontal and vertical directions; (m-t) combinations of various dimensions (major axis length at 18, 24, and 30 km) and velocity reductions (-22%, -27%, and -32%). Bottom panels display the corresponding synthetic RSTFs

as color-shaded waveforms, along with the observed RSTFs shown as black traces, within the frequency range of 0.2-0.8 Hz. The labels in the top panels correspond to the observed RSTFs depicted in the bottom panels.

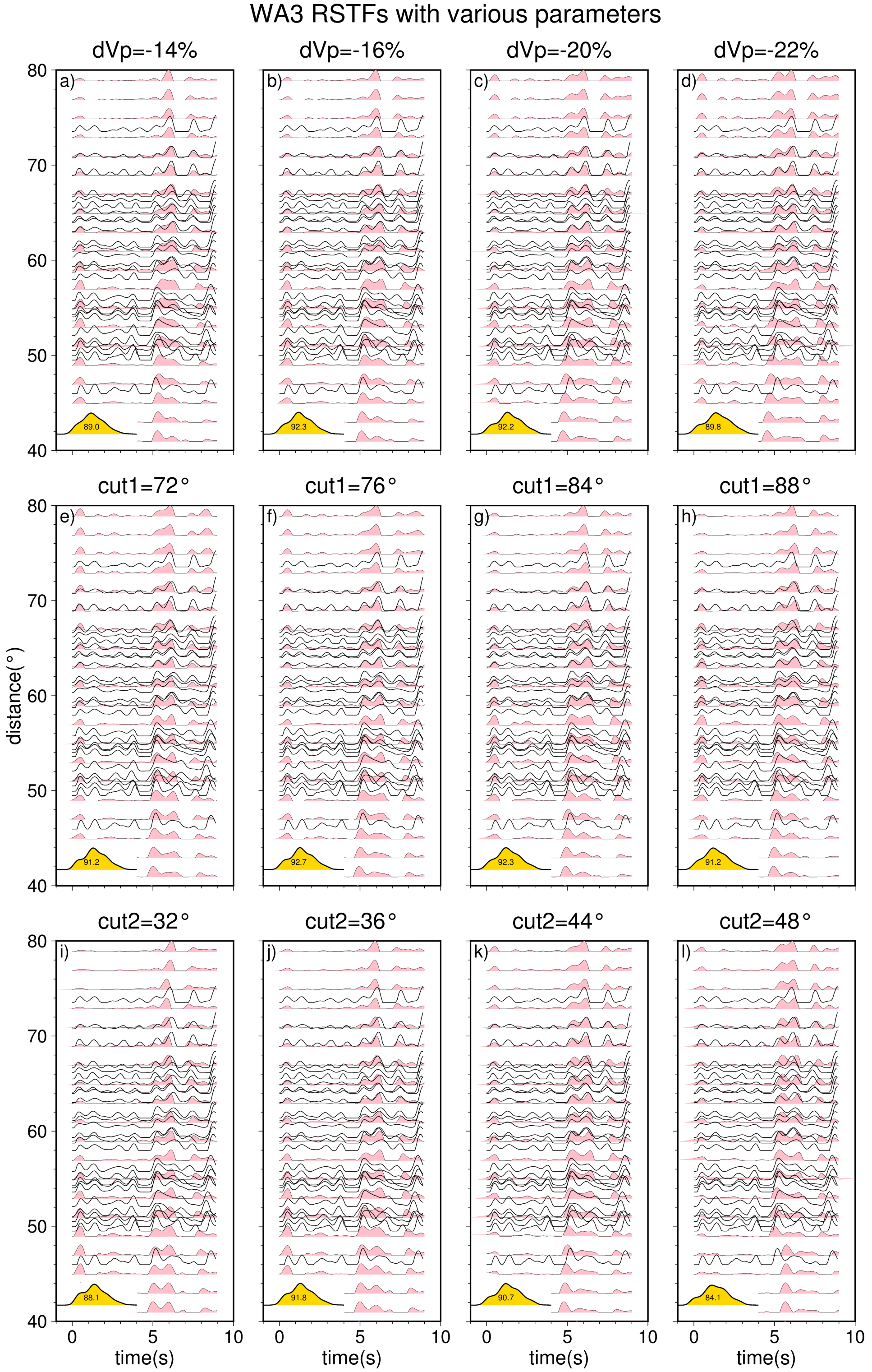

Supplementary Figure 7| Synthetic RSTFs derived from 12 cut-donut models, with each panel corresponding to a variation of one parameter from the optimal model: (a-d) Illustrate variations in P velocity reduction; (e-h) Depict different cutting angles of the left boundary surface of the cut-donut model; (i-l) Represent dipping angles for cutting the right boundary of the model. The color-shaded waveforms in each panel represent the synthetic RSTFs obtained. Additionally, the lower-left corner of each panel shows the TMCC curves in gold, with the corresponding maximum value displayed within.

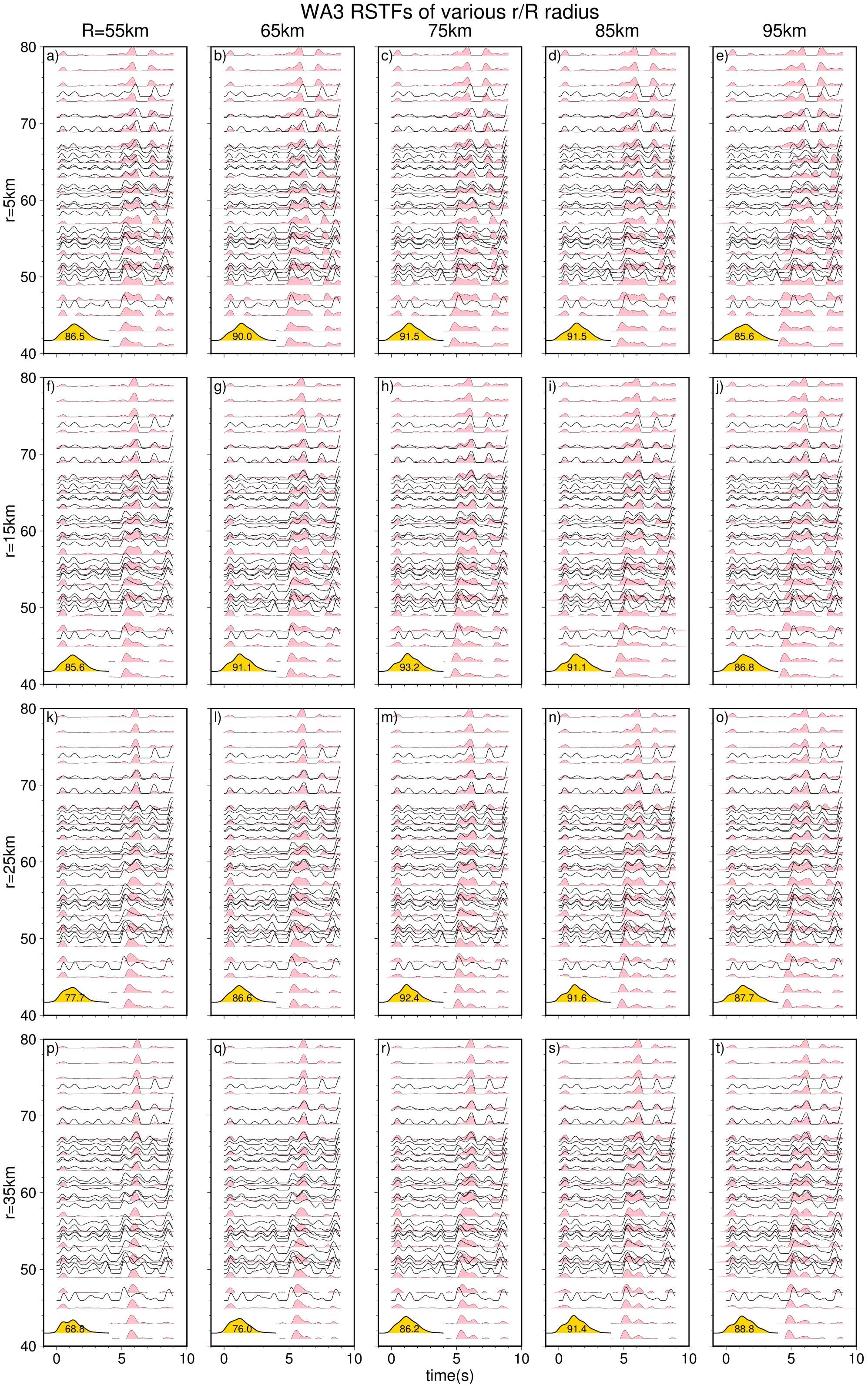
WA3 RSTFs of various r/R radius
R=55km
65km
75km
85km
95km
r=5km
r=15km
r=25km
r=35km
a)
b)
c)
d)
e)
f)
g)
h)
i)
j)
k)
l)
m)
n)
o)
p)
q)
r)
s)
t)
86.5
90.0
91.5
91.5
85.6
85.6
91.1
93.2
91.1
86.8
77.7
86.6
92.4
91.6
87.7
68.8
76.0
86.2
91.4
88.8
80
70
60
50
40
0
5
10
time(s)

Supplementary Figure 8| Synthetic RSTFs derived from variations in two radius values, r and R, in the cut-donut models. Each panel corresponds to a specific combination of radius values. The panels show the color-shaded RSTFs for each combination of radius values, resulting in distinct shapes and characteristics of the RSTFs. In the lower-left corner of each panel, the TMCC curves are displayed in gold, with the corresponding maximum value indicated inside.

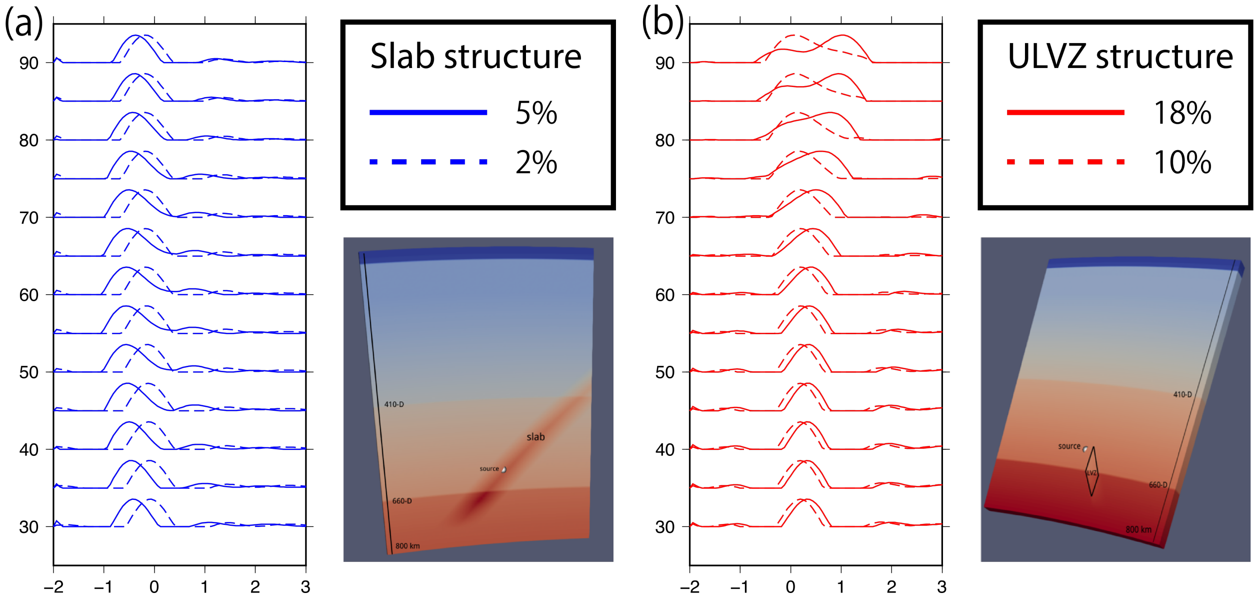

Supplementary Figure 9 | RSTFs derived from 3-D synthetics using hybrid numerical method [6]. This figure showcases the Relative Source Time Functions (RSTFs) obtained from 3-D synthetics generated using a hybrid numerical method. The wavefields were calculated at the boundaries of a 3-D low velocity zone (LVZ) structure using the Direct Solution Method (DSM) to simulate displacement at teleseismic distances [7,8]. Panel (a) displays an 80-km thick slab model with a symmetric positive velocity perturbation concentrated at the core of the subduction. The seismograms in the panel illustrate the effects of 5% and 2% P velocity increments, represented by solid and dashed lines, respectively. Despite the maximum velocity perturbation of 5% penetrating through the 660-D, the slab model only slightly broadens the RSTF without producing a clear multi-pathing "double-peak" feature. In panel (b), a parallelepiped-shaped 3-D low velocity zone (LVZ) structure is positioned along the teleseismic ray path beneath the source, with sharp boundaries. The seismograms in this panel demonstrate the impact of an 18% and 10% P velocity drop, represented by solid and dashed lines, respectively. The 3-D LVZ structure successfully generates multipathing waveforms within the expected distance range, indicating the presence of distinct double-peak features associated with multi-

pathing. Overall, the 3-D synthetics obtained through the hybrid numerical method provide valuable insights into the influence of different velocity perturbations and structures on the resulting RSTFs.

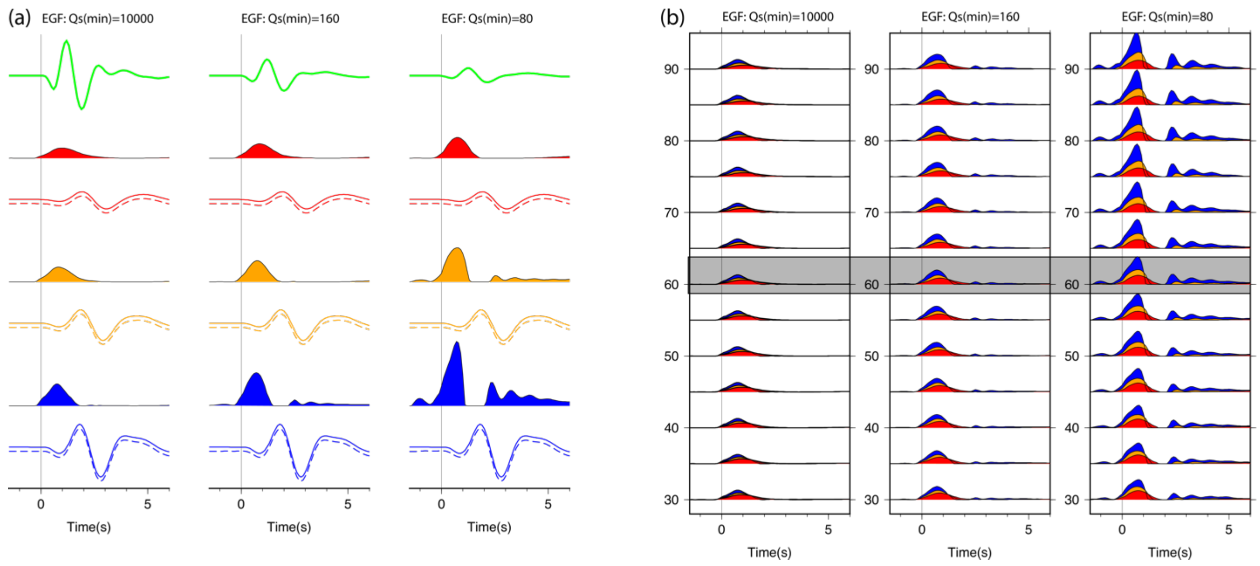

Supplementary Figure 10 | Effect of attenuation on PLD. This figure examines the impact of attenuation on deconvolution by presenting the results of PLD at different distances. Three models with varying levels of attenuation are considered: minimal attenuation (Qp(min) = 20000, Qs(min) = 10000), moderate attenuation (Qp(min) = 320, Qs(min) = 160), and strong attenuation (Qp(min) = 160, Qs(min) = 80).(a) The PLD results at a distance of 60° are shown, generated from synthetics obtained using the different attenuation models. The source with a duration of 0.5 s is located at a depth of 600 km. These synthetics are then treated as EGF for the PLD process. By applying the PLD between the three data sets and three EGF sets, nine RSTFs are obtained at 0.2 - 0.8 Hz, represented by the color-filled waveforms. The data generated by structures with strong, moderate, and no attenuation are colored red, orange, and blue, respectively. The solid and dashed traces represent the filtered data and convolved synthetics, respectively. (b) shows the same analysis as panel (a), but for the entire teleseismic distance profile.

(a)

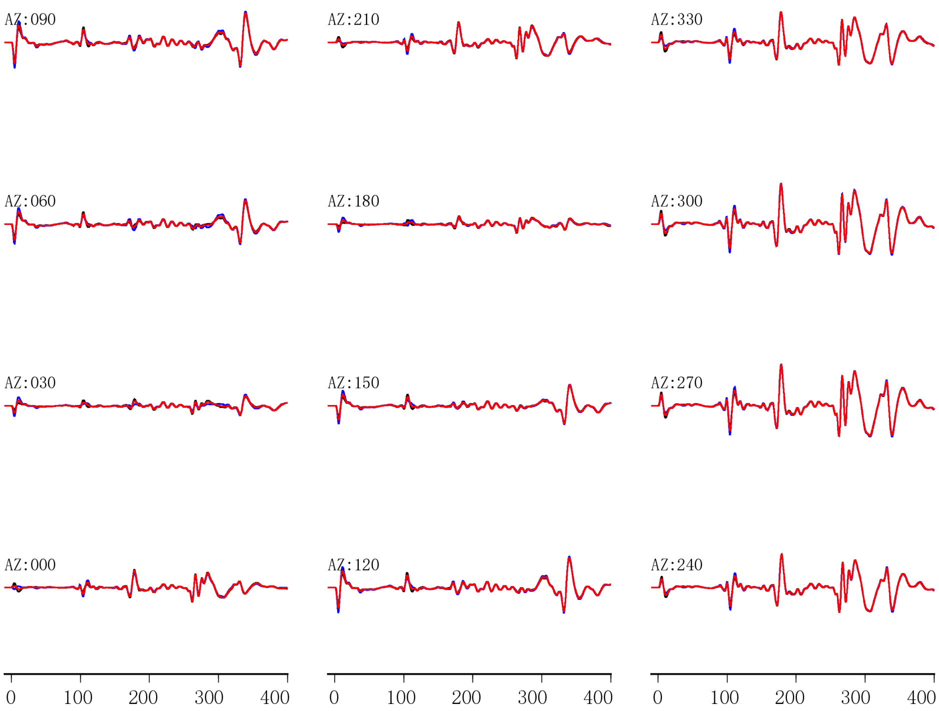
AZ:090
AZ:210
AZ:330
AZ:060
AZ:180
AZ:300
AZ:030
AZ:150
AZ:270
AZ:000
AZ:120
AZ:240
0
100
200
300
400

(b)

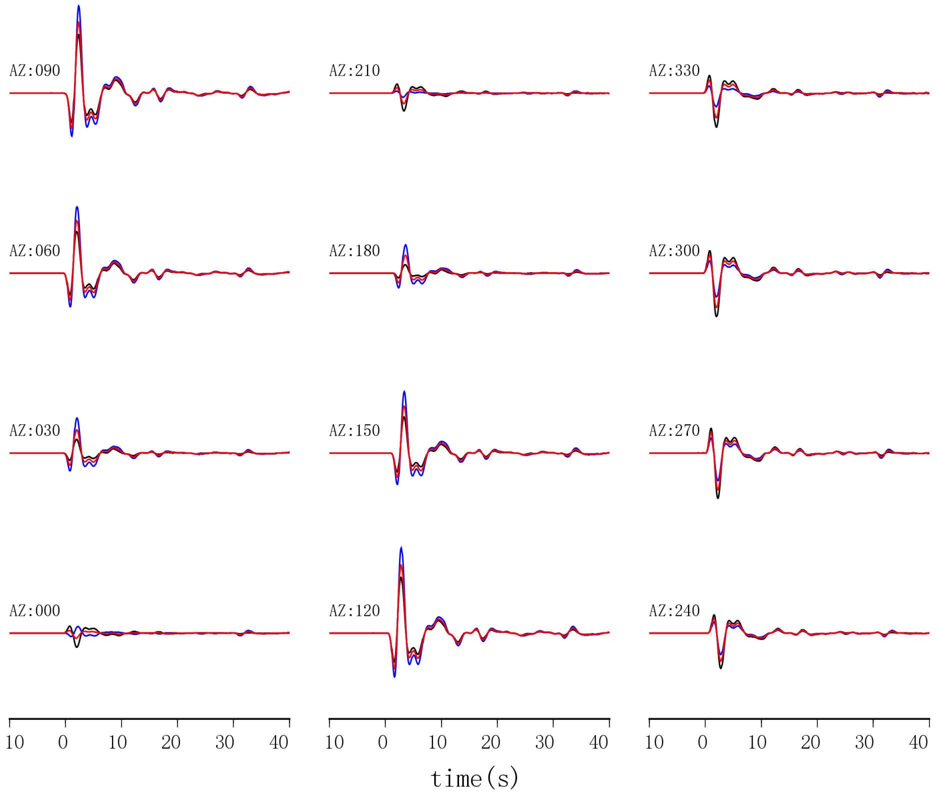
AZ:090
AZ:210
AZ:330
AZ:060
AZ:180
AZ:300
AZ:030
AZ:150
AZ:270
AZ:000
AZ:120
AZ:240
10
0
10
20
30
40
time(s)

Supplementary Figure 11 | Effect of anisotropy on deconvolution. (a) 3-D SEM (Spectral Element Method) synthetics are generated using different J/A values for P waves recorded at an epicentral distance of 30° . Here, J represents one of the 21-parameter weak anisotropy earth models defined by [12], and A represents one of the five elastic parameters of the transversely isotropic model defined in [3]. A 10 km-thick anisotropy layer is placed on top of a 90 km-thick slab to mimic the anisotropic structure for anisotropy model of subduction systems proposed by Li et al., [10]. The waveforms are filtered within the range of 0.01-0.1 Hz, with red traces representing a J/A value of 0.05 and blue traces representing a J/A value of 0.1. (b) Same analysis as in (a) is performed, but with a bandpass filter applied to the waveforms within the range of 0.1-0.5 Hz.

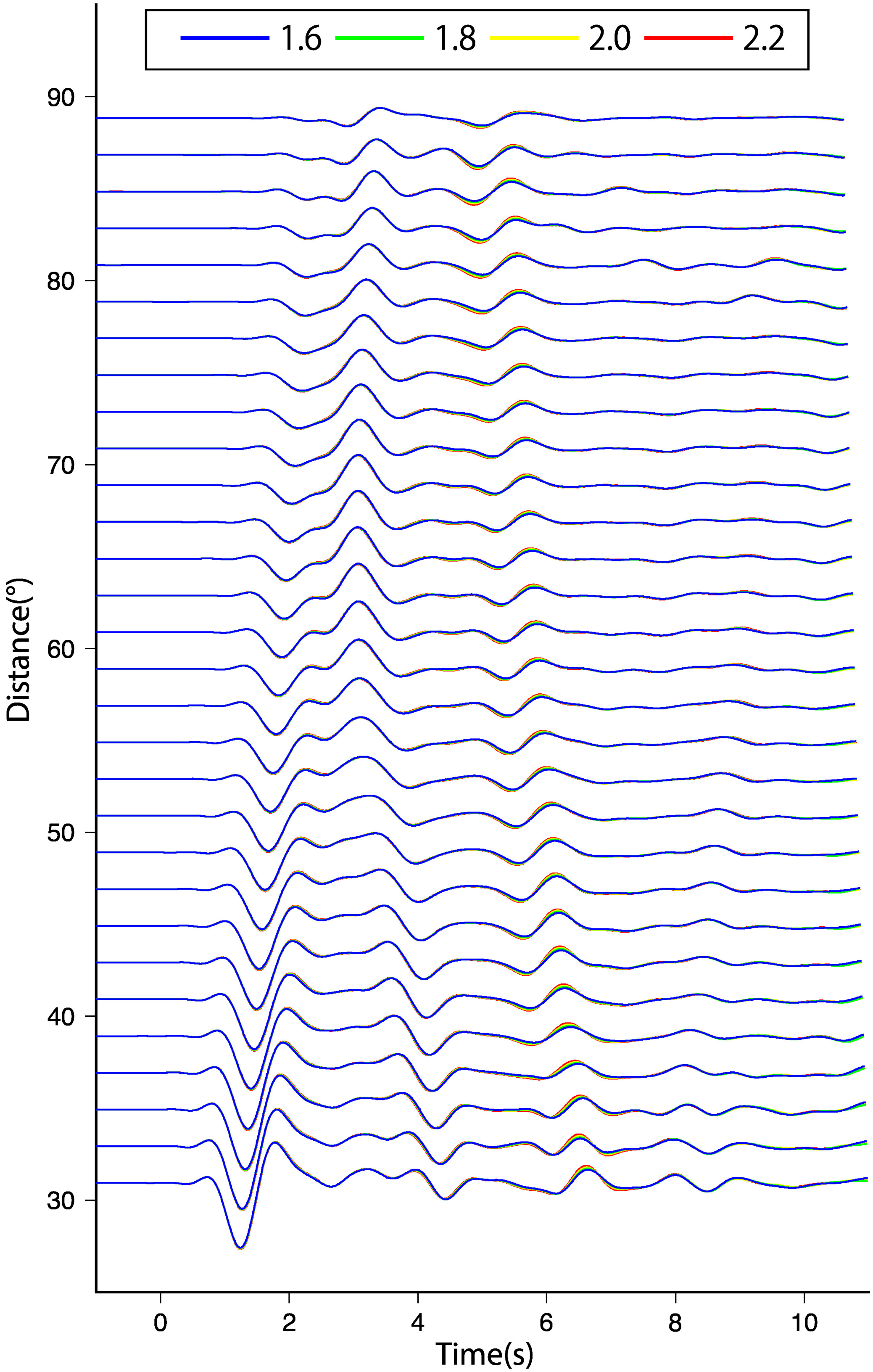

Supplementary Figure 12 | 2-D synthetic waveform colored with different dlnVs/dlnVp of the SULVA model. This figure explores the impact of different values of dlnVs/dlnVp on the synthetic P waveform. A 2-D

synthetic waveform is generated, and the values of dlnVs/dlnVp range from 1.6 to 2.2. The P waveform for the initial 10 seconds remains almost identical across all teleseismic distances.

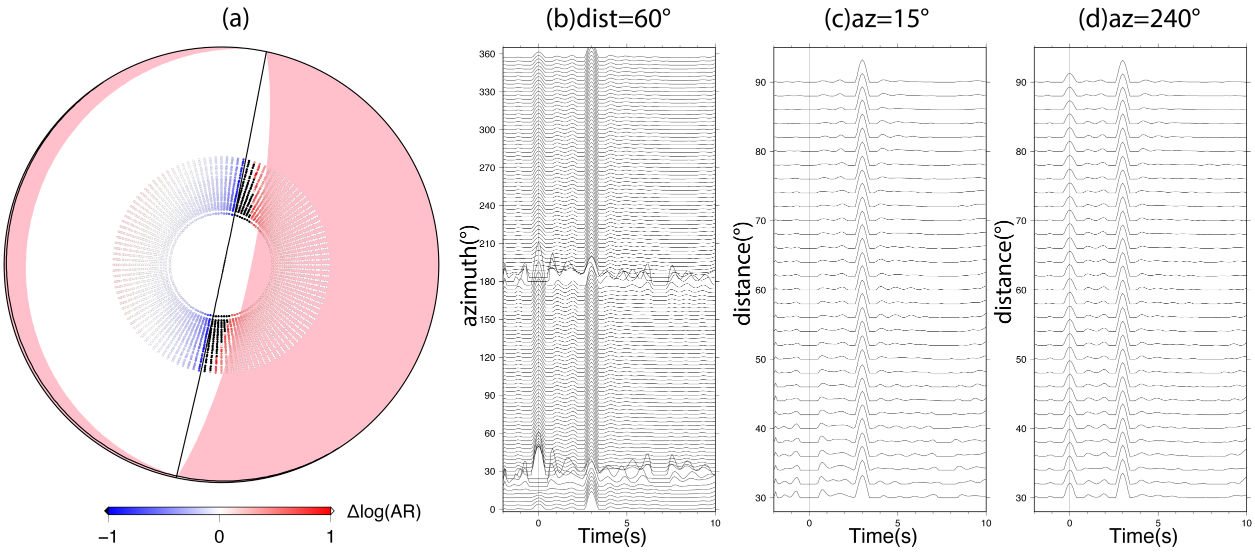

Supplementary Figure 13 | Impact of focal mechanism (FM) variation on deconvolution results: (a) The original globalCMT FM used for EGF is represented by a colored (pink) beach ball, while the solid line depicts a biased FM with a 10° steeper dip. Pseudo-data is generated using the biased FM at 0 seconds and the original FM at 3 seconds. Following the deconvolution process, the logarithmic values of the amplitude ratio between the two subevents are compared to the input value of log(0.5). The resulting differences (Δlog(AR)) are visually indicated by coloring the corresponding incident points along the teleseismic ray path. (b) RSTFs at 60° distance are shown. Notably, the first subevent experiences significant distortion at azimuths of 30° and 180°, which are near the nodal plane of the first FM. (c) A distance profile presented for an azimuth of 15°, where the first subevent exhibits a low amplitude. (d) Another distance profile depicted for an azimuth of 240°. Although the amplitude of the first subevent shows some variations, it remains generally consistent along the profile. Importantly, no multi-pathing effect is observed in both distance profiles.

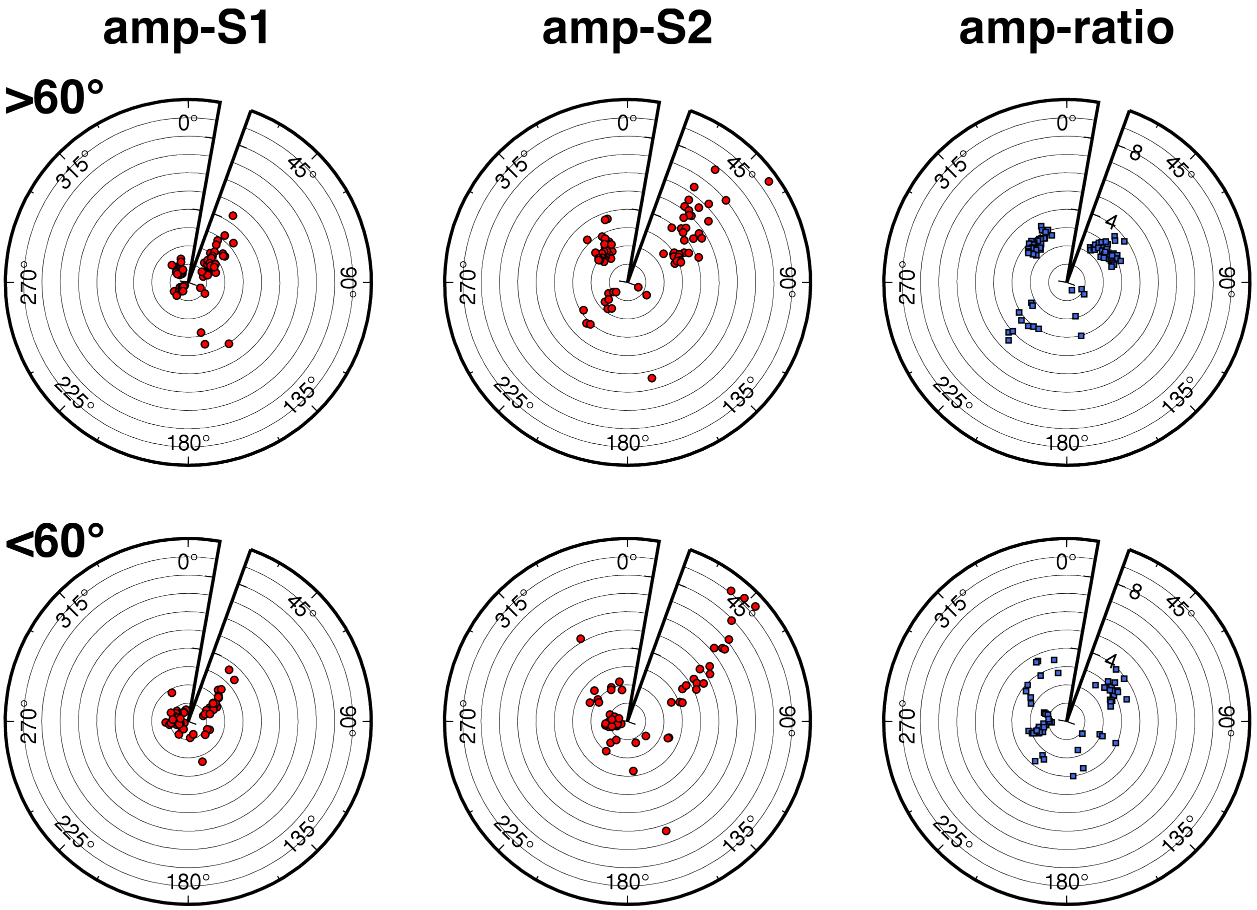

Supplementary Figure 14 | Relative amplitudes of subevents S1 and S2 and their ratios from RSTF stacks in Figure 3(e).

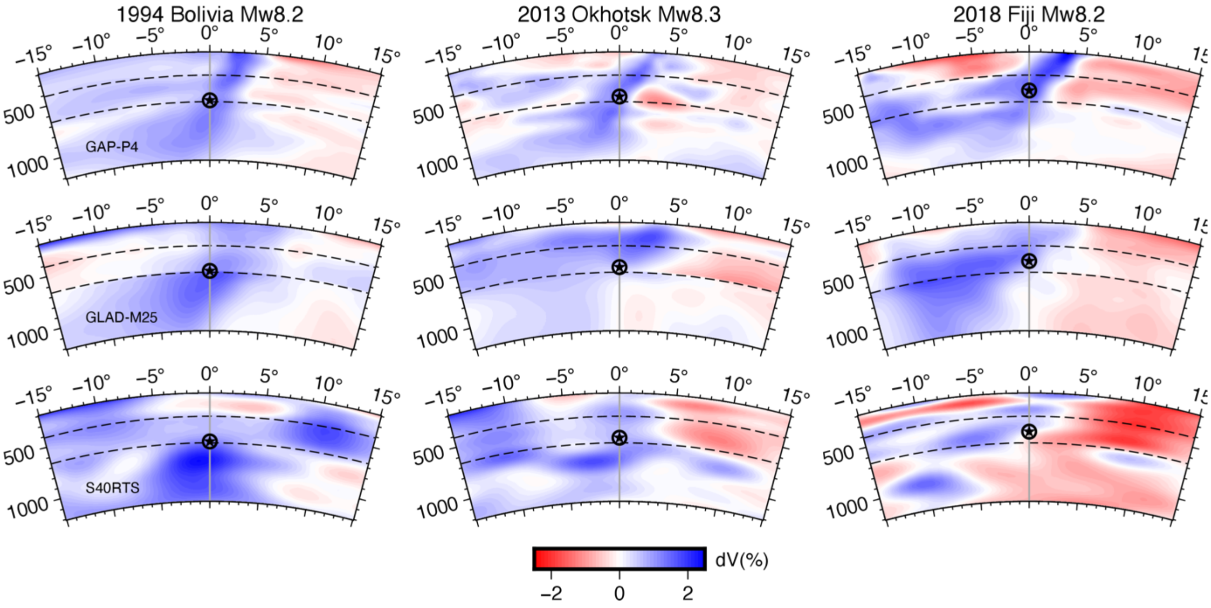

Supplementary Figure 15 | Vertical profiles depicting wave speed anomalies for three tomographic models: P-wave anomalies for GAP-P4 (top) and GLAD-M25 (middle), and S-wave anomalies for S40RTS (bottom) in the context of the three largest deep-seated seismic events. Black stars enclosed by circles denote the centroid locations of these events.

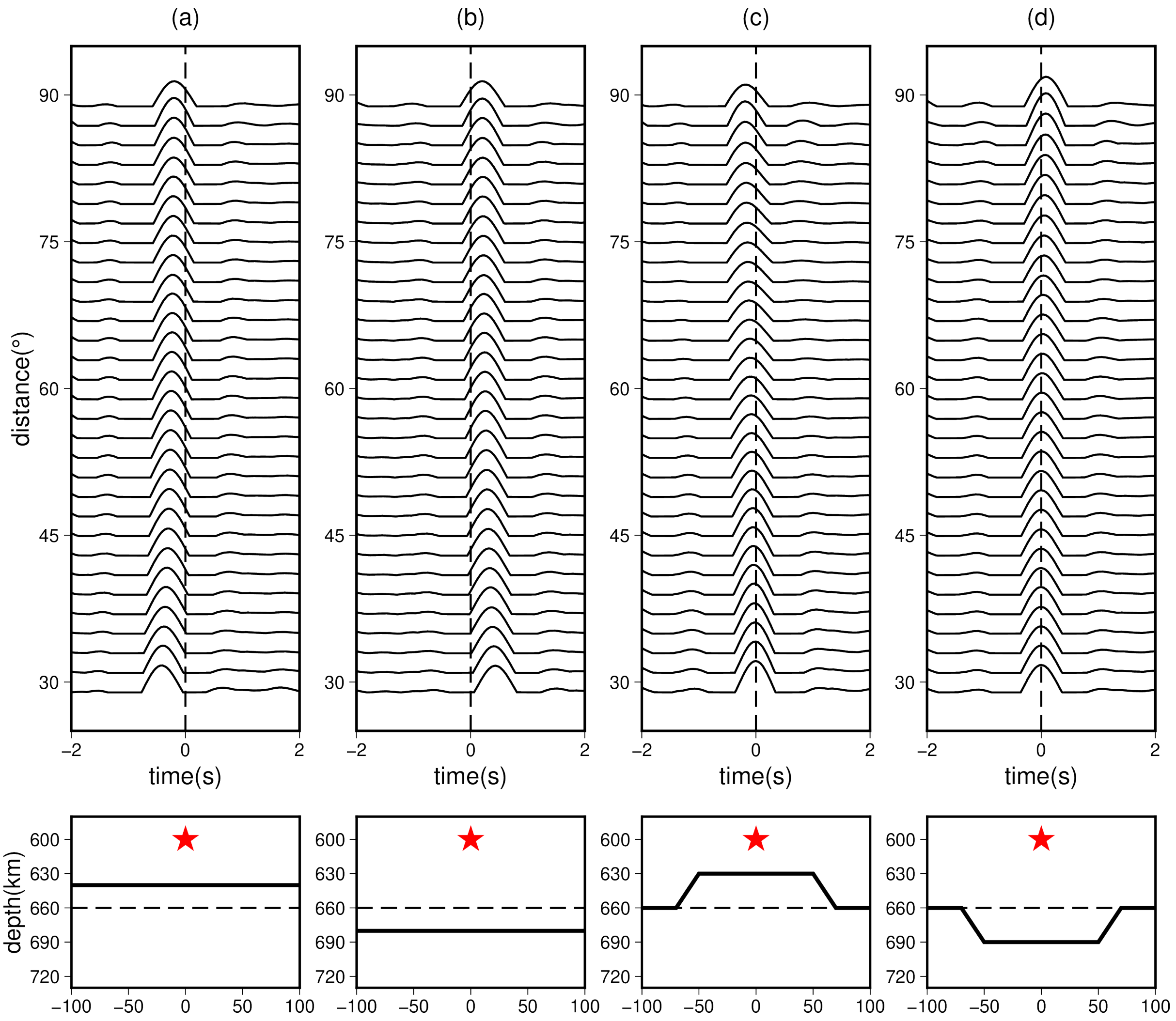

Supplementary Figure 16 | RSTFs obtained under different topographical conditions at 660-D: (a) an elevation of 20 km, (b) a subsidence of 20 km, (c) an elevation with a trapezoid-shaped profile reaching 30 km, and (d) a subsidence with a trapezoid-shaped profile descending 30 km. It is noteworthy that these RSTFs exhibit minimal time shifts and show no substantial waveform distortion. The red star denotes the earthquake source locations, while the thick black lines represent the boundary between the upper and lower mantle.

Supplementary Table 1 Inverted spatial and temporal parameters of subevents Sx and S2

	Vertical offset *H* (km)	Horizontal offset *L* (km)	azimuth Θ (°)	Delayed time τ (s)
**Sx**	2.0 ± 2.6	10.5 ± 0.4	85 ± 2.5	1.70 ± 0.20
**S2**	14.5 ± 2.6	3.1 ± 0.5	51 ± 7.5	2.71 ± 0.20

Supplementary Table 2 | Parameters of optimal "cut-donut" SULVA structure for 5 distance profiles in north America

SUB ARRAY	Azimuth range (°)	cut1(°)	cut2(°)	r (km)	R (km)	dVp (%)
**EA2**	42~50	85	55	10	55	-20
**WA1**	50~55	88	46	15	75	-18
**WA2**	55~60	84	40	20	75	-18
**WA3**	60~68	80	40	15	75	-18
**WA4**	68~74	90	40	15	75	-18

Supplementary References

1. Zhu, L. & Helmberger, D. Advancement in source estimation techniques using broadband regional seismograms. *Bull. Seismol. Soc. Am.* **86**, 1634–1641 (1996).
2. Zhan, Z., Helmberger, D. V., Kanamori, H. & Shearer, P. M. Supershear rupture in a Mw 6.7 aftershock of the 2013 Sea of Okhotsk earthquake. *Science (80-.).* **345**, 204–207 (2014).
3. Dziewonski, A. M. & Anderson, D. L. Preliminary reference Earth model. *Phys. Earth Planet. Inter.* **25**, 297–356 (1981).
4. Sun, D., Helmberger, D., Ni, S. & Bower, D. Direct measures of lateral velocity variation in the deep Earth. *J. Geophys. Res. Solid Earth* **114**, 1–18 (2009).
5. Sun, D., Miller, M. S., Piana Agostinetti, N., Asimow, P. D. & Li, D. High frequency seismic waves and slab

structures beneath Italy. *Earth Planet. Sci. Lett.* **391**, 212–223 (2014).

6. Wu, W., Ni, S., Zhan, Z. & Wei, S. An SEM-DSM three-dimensional hybrid method for modelling teleseismic waves with complicated source-side structures. *Geophys. J. Int.* **215**, 133–154 (2018).
7. Geller, R. J. & Takeuchi, N. A new method for computing highly accurate DSM synthetic seismograms. *Geophys. J. Int.* **123**, 449–470 (1995).
8. Okamoto, T. Teleseismic synthetics obtained from 3-D calculations in 2-D media. *Geophys. J. Int.* **118**, 613–622 (1994).
9. Karaoğlu, H. & Romanowicz, B. Inferring global upper-mantle shear attenuation structure by waveform tomography using the spectral element method. *Geophys. J. Int.* **213**, 1536–1558 (2018).
10. Li, J., Zheng, Y., Thomsen, L., Lapen, T. J. & Fang, X. Deep earthquakes in subducting slabs hosted in highly anisotropic rock fabric. *Nat. Geosci.* **11**, 696–700 (2018).
11. Komatitsch, D. & Tromp, J. Introduction to the spectral element method for three-dimensional seismic wave propagation. *Geophys. J. Int.* **139**, 806–822 (1999).
12. Chen, M. & Tromp, J. Theoretical and numerical investigations of global and regional seismic wave propagation in weakly anisotropic earth models. *Geophys. J. Int.* **168**, 1130–1152 (2007).
13. Williams, Q. & Garnero, E. J. Seismic evidence for partial melt at the base of earth's mantle. *Science (80-.).* **273**, 1528–1530 (1996).
14. McNamara, A. K. A review of large low shear velocity provinces and ultra low velocity zones. *Tectonophysics* **760**, 199–220 (2019).
15. Takei, Y. Effect of pore geometry on V P / V S : From equilibrium geometry to crack. *J. Geophys. Res.* **107**, ECV 6-1 (2002).
16. Schmandt, B., Dueker, K. G., Hansen, S. M., Jasbinsek, J. J. & Zhang, Z. A sporadic low-velocity layer atop the western U.S. mantle transition zone and short-wavelength variations in transition zone discontinuities. *Geochemistry, Geophys. Geosystems* **12**, (2011).
17. Jasbinsek, J. & Dueker, K. Ubiquitous low-velocity layer atop the 410-km discontinuity in the northern Rocky Mountains. *Geochemistry, Geophys. Geosystems* **8**, 1–19 (2007).
18. Zhan, Z., Kanamori, H., Tsai, V. C., Helmberger, D. V. & Wei, S. Rupture complexity of the 1994 Bolivia and 2013 Sea of Okhotsk deep earthquakes. *Earth Planet. Sci. Lett.* **385**, 89–96 (2014).
19. Chen, Y., Wen, L. & Ji, C. A cascading failure during the 24 May 2013 great Okhotsk deep earthquake. *J. Geophys. Res. Solid Earth* **119**, 3035–3049 (2014).
20. Bertero, M. *et al.* Application of the projected Landweber method to the estimation of the source time function in seismology. *Inverse Probl.* **13**, 465–466 (1997).
